# Exosomal lncRNAs *HOTAIR*, *HULC*, and *ANRIL*: Decoding the Biomarker Landscape of Sporadic Triple-Negative Breast Cancer

**DOI:** 10.3390/ijms27104308

**Published:** 2026-05-12

**Authors:** Hazal Sezginer Guler, Hakan Gurkan, Sinem Yalcintepe, Yavuz Atakan Sezer, Sernaz Topaloglu, Ebru Tastekin, Selcuk Korkmaz, Daghan Dagdelen, Engin Atli, Selma Demir, Nermin Tuncbilek

**Affiliations:** 1 Departmant of Medical Genetics, Faculty of Medicine, Trakya University, Edirne 22030, Turkey; hgurkan@trakya.edu.tr (H.G.); sinemyalcintepe@trakya.edu.tr (S.Y.); enginatli@trakya.edu.tr (E.A.); selmaulusal@trakya.edu.tr (S.D.); 2 Departmant of General Surgery, Faculty of Medicine, Trakya University, Edirne 22030, Turkey; atakansezer@trakya.edu.tr; 3 Departmant of Oncology, Faculty of Medicine, Trakya University, Edirne 22030, Turkey; sernazuzunoglu@trakya.edu.tr; 4 Departmant of Pathology, Faculty of Medicine, Trakya University, Edirne 22030, Turkey; ebrutastekin@trakya.edu.tr; 5 Departmant of Biostatistics, Faculty of Medicine, Trakya University, Edirne 22030, Turkey; selcukkorkmaz@trakya.edu.tr; 6 Department of Plastic Surgery, Faculty of Medicine, Marmara University, Istanbul 34854, Turkey; daghan.dagdelen@marmara.edu.tr; 7 Departmant of Radiology, Faculty of Medicine, Trakya University, Edirne 22030, Turkey; ntuncbilek@trakya.edu.tr

**Keywords:** triple-negative breast cancer (TNBC), sporadic breast cancer, long non-coding RNAs (lncRNAs), exosomal RNA, biomarker, prognostic marker

## Abstract

Triple-negative breast cancer (TNBC/18–21%) lacks targeted treatment options due to the lack of ER/PR and HER2 expression. The transport of lncRNAs via exosomes plays a role in tumor progression and metastasis and reshapes tumor-associated signaling pathways. This study aimed to compare the expression of exosomal *HOTAIR*, *NEAT1*, *MALAT1*, *AFAP1-AS1*, *ANRIL*, and *HULC* lncRNAs in primary tumor tissue and blood of patients with sporadic TNBC to evaluate their potential as biomarkers. The patients diagnosed with TNBC between the years 2021 and 2025, 21 of 62 (33.87%) with sporadic breast cancer and thirty healthy controls were included in the study. Primary tumor tissue and peripheral venous blood samples were collected from 21 patients who did not receive neoadjuvant chemotherapy. Expression levels of exosomal lncRNAs (*HOTAIR*, *NEAT1*, *MALAT1*, *AFAP1-AS1*, *ANRIL*, and *HULC*) were determined in both tissues and blood samples from the patient and control groups using Real-Time PCR method. In the patient group, *HOTAIR*, *HULC*, *ANRIL*, and *AFAP1_AS1* gene expression was lower (downregulated) in tissue and serum compared to the control group, whereas *NEAT1* and *MALAT1* were higher (upregulated). Tissue and serum samples taken from the patient group were found to have statistically consistent expression levels of *HOTAIR*, *HULC*, and *ANRIL* genes. Furthermore, *HOTAIR*, *HULC*, and *ANRIL* serve as biomarkers and can be studied using exosomal RNA samples obtained from patient serum without invasive procedures. Our current study, which has different lncRNA expression profiles, reflects the biological heterogeneity of TNBC and contributes to a better understanding of its subtypes at the molecular level.

## 1. Introduction

Breast cancer is the most common type of cancer worldwide, particularly among women. While advances in screening and treatment have reduced the overall risk of death from the disease, the number of people diagnosed with breast cancer continues to increase. Data have shown that the incidence of breast cancer has increased by 1% annually since 2012. Current estimates (American Cancer Society) predict that by 2025, approximately 316,950 women will be diagnosed with invasive breast cancer, approximately 16% of whom will be under the age of 50. Breast cancer is a highly molecularly heterogeneous group and is divided into subtypes based on gene expression. Triple-negative breast cancer (TNBC), which has limited treatment options, lacks ER/PR (estrogen receptor/progesterone receptor) and HER2 (human epidermal growth factor receptor 2) expression and accounts for 18–21% of invasive breast cancers [1]. Furthermore, the percentage of Ki67 protein is also emphasized as being crucial for metastasis [2].

Approximately 10–15% of breast cancer cases are familial and associated with known gene variants. Studies in TNBC are critical today, especially considering the high percentage of individuals with sporadic breast cancer. The lack of gene expression markers in the TNBC molecular classification limits the effectiveness of patient treatment strategies. Therefore, there is a need to identify more genetically based biomarkers.

Biomarkers can be analyzed in various human samples, including tissue, blood, urine, and other body fluids. Currently, for breast cancer, CA15-3 and CEA (*CA15-3: metastatic breast cancer marker, *CEA: carcinoembryonic antigen) are commonly analyzed in blood samples [3]. Genetically, biomarkers can be derived from the type of sample to be investigated and from various biological materials thought to be influential in the cancer development process. In recent biomarker research, exosomes, the fundamental elements of intercellular interactions, have become prominent, rather than DNA and RNA obtained from basic biological samples.

Exosomes are lipid-bilayered extracellular vesicles, 30–150 nm in size, released by all cell types, including breast cancer cells. Their vesicle contents consist of proteins, lipids, metabolites, DNA, mRNA, and non-coding RNAs (short miRNAs and long non-coding RNAs (lncRNAs). Cancer cells utilize exosomes to achieve metastasis, immune evasion, drug resistance, immune suppression, communication with the tumor microenvironment, and invasion. Exosomes facilitate tumor progression and metastasis by carrying oncogenic signaling proteins, ligands, enzymes, and non-coding RNAs on their surfaces or within their vesicles [4]. Furthermore, studies on TNBC suggest that exosomal lncRNAs regulate gene expression in the nucleus and cytoplasm and interact with multiple post-transcriptional mechanisms [5].

LncRNAs regulate intracellular and intercellular signaling in TNBC. Depending on their mechanisms, they can affect gene expression in multiple ways and, consequently, transform cells into a cancer phenotype [6]. Studies generally reveal differences in lncRNA expression levels between patient and healthy tissue samples, underscoring their potential as biomarkers [7]. Biomarker studies typically focus on the potential of a single lncRNA in different sample types [8]. Meta-analysis of international data indicates that lncRNAs with high expression levels are positively correlated with lymph node metastasis and distant metastasis [9].

**Figure 1:**
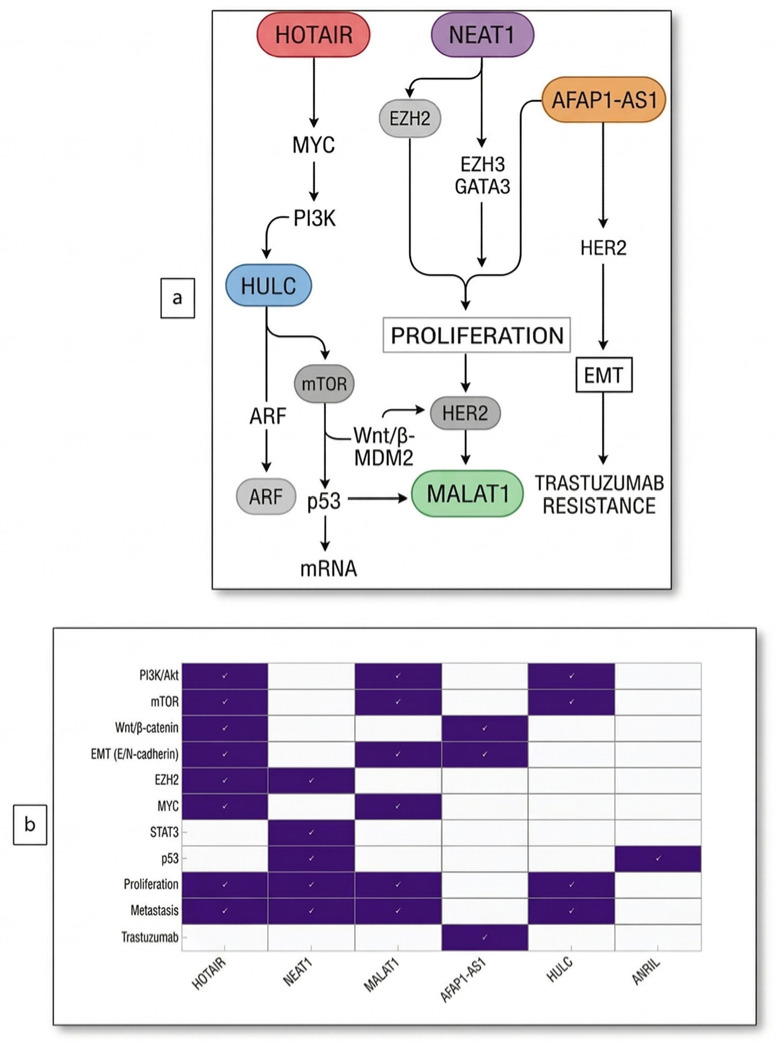
(**a**) Effects of lncRNAs investigated in the current study on common cellular signaling pathways, (**b**) Molecular targets and locations at common pathway intersections of lncRNAs were investigated in the current study.

According to *GENCODE 24 data, 21,000 lncRNAs have been identified to date. Recent studies have highlighted 51 lncRNAs as crucial for breast cancer. Our current study identified six lncRNAs that we predict are associated with TNBC and play regulatory roles in intracellular and intercellular signaling during cancer development. The common cellular signaling pathways and effects of the lncRNAs investigated in this study are presented in Figure 1.

The primary hypothesis of our study was to investigate the potential use of lncRNAs as biomarkers in sporadic TNBC. The paucity of molecular markers in TNBC complicates early diagnosis and prognosis, limiting treatment strategies. Because lncRNAs transported via exosomes are known to play an active role in tumor progression and metastasis, examining serum levels of these molecules could reveal the biological heterogeneity of TNBC through a non-invasive approach. In particular, downregulation of *HOTAIR*, *HULC*, and *ANRIL*, and upregulation of *NEAT1* and *MALAT1*, reflect the TNBC-specific molecular signature and may guide clinical practice in identifying diagnostic, prognostic, and therapeutic targets. Furthermore, we demonstrate that the *HOTAIR*, *HULC*, and *ANRIL* lncRNAs investigated in this study are present at consistent levels in both tissue- and serum-derived exosomal RNAs. This finding reveals that these genes are strong biomarker candidates and can be evaluated through serum samples without the need for invasive biopsy.

## 2. Results

Of the 582 patients initially diagnosed with breast cancer, 21 of 62 TNBC patients with sporadic cases were included in the study according to exclusion criteria. A germline cancer NGS panel was performed on all 62 patients diagnosed with TNBC, and 21 (33.87%) were diagnosed with sporadic breast cancer after no germline pathogenic or likely pathogenic variants were detected. Seven (33.33%) of our patients developed metastasis within the first three years. Additionally, two cases with metastasis were reported to have uterine polyps, one case with a renal cyst, and one case with a thyroid nodule. After receiving their initial diagnosis, the patients in our patient group began combined chemotherapy and adjunctive treatments based on their clinical condition. Since the initiation of our study, we have also determined the recurrence and survival rates of the patients.

The patients included in the patient group were aged between 46 and 55 (mean age: 50.5). This suggests that TNBC is more common in our patient population, particularly in middle-aged women. The majority of patients had a Ki67 proliferation index above 30%. This supports the conclusion that TNBC patients, most of whom were in stage T3, exhibit aggressive biological behavior. This distribution supports the conclusion that most cases were in advanced stages at diagnosis. Nodal metastases (N0) were detected in two-thirds of the cohort, while nodal metastases (N1) were detected in one-third. These patients had poorer survival compared to N0 patients. All patients who received neoadjuvant therapy, carboplatin- and taxol-based regimens, and pembrolizumab are currently under follow-up and alive. The higher survival rate in carboplatin-containing protocols is noteworthy.

The four-year survival rate from diagnosis and treatment initiation to the present day was calculated using the Kaplan–Meier method. Five of the 21 patients included in the study died during this period. Since 16 patients were alive at the end of four years, the survival rate was 16/21 ≈ 0.76 (76.20%). Because TNBC is a more aggressive subtype of breast cancer than other breast cancer subtypes, the risk of recurrence is higher in individuals who respond positively after the first course of treatment. According to our data over the four years, 15 of the 21 patients (71.42%) experienced recurrence, with 12 occurring within the first two years.

Our criteria for comparing lncRNA expression levels between groups in sporadic TNBC patients, which constitute the hypothesis of our study, are as follows: patient group tissue–control group tissue data, patient group serum–control group serum data, patient group tissue–patient group serum data, control group tissue–control group serum data, patient group metastasis status–lncRNA expression levels, patient group cancer markers in blood–lncRNA expression levels.

**Figure 2:**
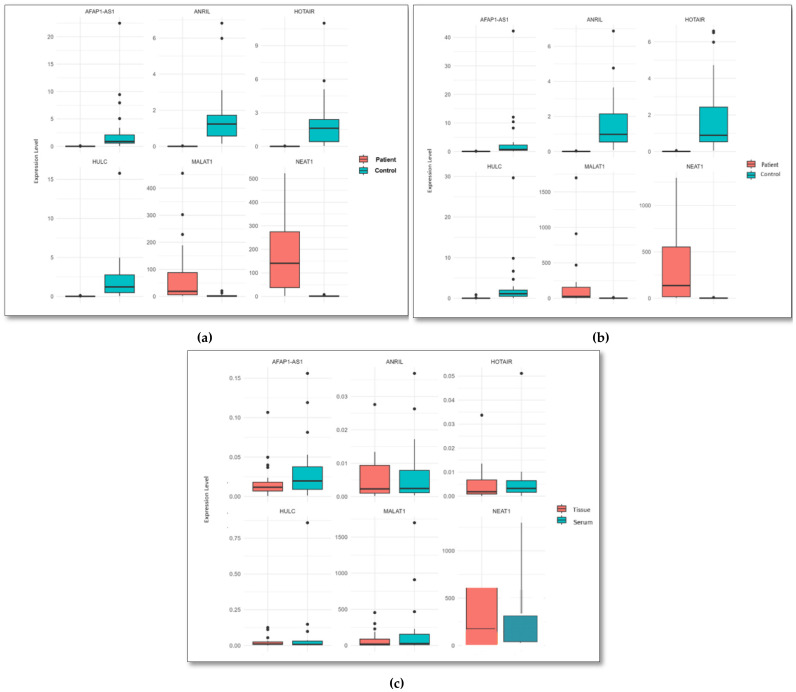
(**a**) Comparison of gene expression of long non-coding RNAs between patient and control group tissue samples, (**b**) Comparison of gene expression of long non-coding RNAs between patient and control group serum samples, (**c**) Comparison of gene expression of long non-coding RNAs between patient group tissue and serum sample.

When the tissue data from the patient and control groups were statistically compared, it was found that *HOTAIR*, *HULC*, *ANRIL*, and *AFAP1_AS1* were downregulated in the patient group, while *NEAT1* and *MALAT1* were upregulated (Table 1, Figure 2).When the serum data from the patient and control groups were statistically compared, it was found that *HOTAIR*, *HULC*, *ANRIL*, and *AFAP1_AS1* were downregulated in the patient group, while *NEAT1* and *MALAT1* were upregulated (Table 2 and Figure 2). When the tissue and serum samples from the patient group were statistically compared, the expression levels of exosomal *HOTAIR*, *HULC*, and *ANRIL* genes were found to be consistent between the two. Based on this result, we predict that the expression levels of exosomal *HOTAIR*, *HULC*, and *ANRIL* in serum can serve as biomarkers without the need for invasive procedures such as tissue biopsy (Table 3 and Figure 2). The consistency between tissue and serum expression patterns was further evaluated based on the direction of expression changes. *HOTAIR*, *HULC*, and *ANRIL* showed concordant expression patterns in both tissue and serum samples, with consistent downregulation across sample types. In addition, statistical analyses performed in the patient group (Table 3) demonstrated significant differences in expression levels, further supporting the consistency between tissue and serum-derived exosomal lncRNA profiles.

The *NEAT1* gene was more highly expressed in tissue than in serum. The *AFAP1-AS1* and *MALAT1* genes were less expressed in tissue but more so in serum. The relationship between metastasis and the expression levels of related genes in the patient group was evaluated using the *T*-test and Mann–Whitney U test, and the *NEAT1* gene was found to be highly expressed in non-metastatic patients (*p* < 0.0042). No significant differences were found for other genes. Biochemical cancer markers (CEA, CEA 15-3) in the blood were also statistically evaluated using the *T*-test and Mann–Whitney U test. No significant results were found for any of the genes in the *T*-test results. However, an abnormal decrease in *NEAT1* gene expression was observed using the Mann–Whitney U test. When control group tissue and control group serum data were compared, no statistically significant difference was detected.

**Figure 3:**
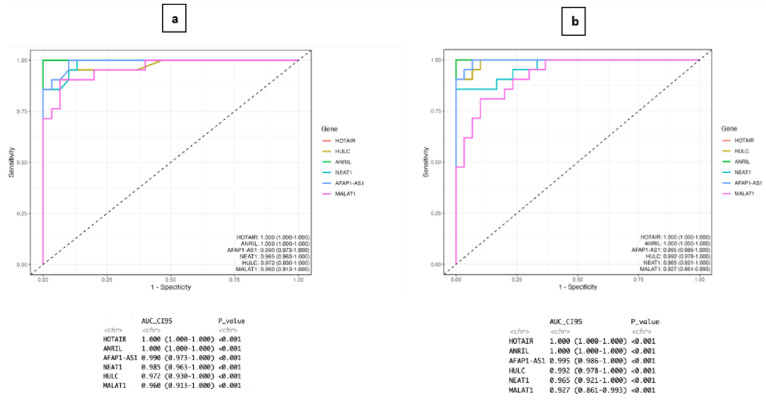
Receiver operating characteristic (ROC) curve analysis of exosomal lncRNAs for distinguishing TNBC patients from healthy controls, (**a**) ROC curves based on serum-derived exosomal lncRNA expression levels, (**b**) ROC curves based on tissue-derived exosomal lncRNA expression levels.

ROC analysis demonstrated that serum exosomal lncRNAs exhibited strong diagnostic performance in distinguishing sporadic TNBC patients from healthy controls. *HOTAIR* and *ANRIL* showed excellent discriminatory power (AUC = 1.000), while AFAP1-AS1, *NEAT1*, *HULC*, and *MALAT1* demonstrated high diagnostic accuracy with AUC values ranging from 0.960 to 0.990 (*p* < 0.001 for all genes). These findings suggest that circulating exosomal lncRNAs may be promising, non-invasive biomarkers for TNBC detection. However, the number of patients included in this study was relatively limited due to technical constraints associated with exosome isolation and subsequent molecular analysis (Figure 3). Furthermore, the analyzed lncRNAs showed strong diagnostic performance in distinguishing TNBC patients from healthy controls. However, these findings should be interpreted with caution due to the relatively small cohort size, and further validation in larger, independent cohorts is required. Overall, our results highlight the potential clinical utility of exosomal lncRNAs as promising biomarkers for TNBC diagnosis and monitoring.

## 3. Discussion

TNBC is characterized by profound biological heterogeneity, which complicates efforts to identify meaningful molecular markers. Our study addresses this gap by comparing exosomal lncRNA expression profiles in tumor tissue and serum from patients with sporadic TNBC. In this context, our study suggests that exosomal lncRNAs, particularly *HOTAIR*, *HULC*, and *ANRIL*, may be promising biomarker candidates. These three lncRNAs showed consistent downregulation in both tumor tissue and serum-derived exosomes, suggesting that circulating exosomal RNA may reflect tumor biology without requiring invasive tissue sampling. On the other hand, increased expression of *NEAT1* and *MALAT1* further underscores their potential contribution to tumor aggressiveness and may aid in identifying subgroups within sporadic TNBC.

Research shows that exosome secretion is higher in individuals with cancer than in healthy individuals. The increased exosome secretion in individuals with cancer may be explained by the activation of the *TSAP6* (metalloreductase) gene, which regulates the exosomal secretion pathway within the p53 pathway [10]. A meta-analysis of international data reported that highly expressed lncRNAs are positively correlated with lymph node metastasis and distant metastasis. Our study identified the lncRNAs *HOTAIR*, *NEAT1*, *AFAP1-AS1*, *ANRIL*, *MALAT1*, and *HULC*, which are linked to metastasis. These lncRNAs are prominent not only in metastasis but also in various mechanisms during the cancerization process of TNBC cells.

To summarize the results of our study, the downregulated lncRNAs were *HOTAIR*, *HULC*, *ANRIL*, and *AFAP1_AS1*, while the upregulated lncRNAs were *NEAT1* and *MALAT1*. Studies reported in the literature generally only examined expression levels in cell lines, tumor tissue, or serum/plasma samples. Studies that combined or compared all parameters are almost nonexistent. Therefore, further studies are needed to enrich the existing data. In our study, the data obtained for *HOTAIR*, *ANRIL*, and *AFAP1_AS1* differ from those reported in the literature. In our study, we specifically investigated lncRNA expression levels in primary tumor tissues and serum samples from sporadic TNBC patients before chemotherapy or radiotherapy.

*HOTAIR*, whose elevated expression has been associated with metastasis in TNBC studies, may also act as a tumor suppressor gene, depending on the pathway in which it functions [11]. Expression levels vary in various subgroups of breast cancer, particularly in tumor tissues. It is known for its increased expression levels in serum samples [12]. In our study, a statistically significant difference in *HOTAIR* expression levels was observed between tissue and serum samples from the patient and control groups. *HOTAIR* expression levels were lower in both tissue and serum samples in the patient group than in the control group. *HOTAIR* expression levels in tissue and serum samples from the patient group were parallel. A study including 2192 individuals with TNBC emphasized that *HOTAIR* was highly expressed in tumor tissue, which may be associated with poor prognosis and metastasis [13]. The number of studies evaluating *HOTAIR* expression in serum samples from TNBC patients is quite limited, and, generally, increased expression has been reported only in breast cancer patients. Li et al. reported that expression levels in tumor tissue may vary with cancer stage [14]. The study emphasized that *HOTAIR* expression levels in stage I breast cancer were lower than in stages II, III, and IV [15]. Another study conducted with a breast cancer cell line reported that *HOTAIR* regulates resistance to radiation and chemotherapy in breast cancer [16]. Another study conducted in 2019 demonstrated the effects of *HOTAIR* expression on treatment resistance in TNBC cells using a recombinant plasmid vector containing *HOTAIR*. This study, conducted in a cell line, reported that resistance to the treatment agent decreased in cell groups with lower *HOTAIR* expression [17]. The results of our study regarding *HOTAIR* expression levels differ from those reported in the literature.

Unlike other lncRNAs, *NEAT1* forms a unique nuclear body (paraspeckles) that facilitates the assembly of various protein components, including RNA-binding proteins. Its association with metastasis is highlighted by its increased expression levels in other breast cancer subgroups, including TNBC. *NEAT1* is regulated by p53 to suppress tumor transformation. *NEAT1* affects aerobic glucose and lactic acid fermentation for energy production in cancer cells. This is also related to the Warburg effect.

*NEAT1* is known for its elevated expression in studies of breast cancer and TNBC. In 2019, Shin et al. reported that *NEAT1* expression levels in blood samples from TNBC patients were higher than in healthy individuals [18]. In another study of 164 TNBC individuals, increased expression levels in tumor tissue were associated with lymph node metastasis, highlighting its utility as a biomarker for patient follow-up and cancer management [19]. Furthermore, in another study involving treatment-resistant cancer stem cells, it was concluded that when *NEAT1* was completely knocked out, chemotherapy-resistant cells became sensitive to the drug, thereby promoting drug uptake [20]. In our study, consistent with the literature, *NEAT1* expression levels were higher in tissue and serum samples from the patient group than in those from the control group. We also found that *NEAT1* expression was higher in tissue samples from the patient group compared to serum samples.

*AFAP1-AS1*, which plays a role in various types of cancer, acts as an oncogene in TNBC cells, increasing metastasis and tumor invasion, and many studies on breast cancer have reported increased expression levels in tissue and serum. In our research, we found that *AFAP1_AS1* was less expressed in the patient group than in the control group. When the patient group was evaluated within the patient group, its expression level in tissue was lower than in serum samples. A study of the TNBC MDA-MB-231 cell line reported high *AFAP1_AS1* expression. It has also been reported that this upregulation may increase the proliferation and invasiveness of TNBC cells [21]. Studies investigating drug resistance mechanisms have reported that certain lncRNAs are upregulated in trastuzumab-resistant breast cancer cells and can be transferred via exosomes, contributing to the dissemination of resistance between cells. For instance, *AFAP1-AS1* has been associated with trastuzumab resistance and may be involved in exosome-mediated intercellular communication, although further validation is required [22]. Our data for *AFAP1_AS1*, which has been frequently reported to show increased expression in previous studies, differ from those studies.

*ANRIL* is involved in the mechanisms of apoptosis, metastasis, invasion, and proliferation in cancer cells via the p53 pathway. It is known that expression levels are higher in TNBC cell lines than in healthy cells. In our study, contrary to the literature, *ANRIL* expression levels were lower in tissue and serum samples from the patient group than in those from the control group. Studies on TNBC in the literature generally include cell lines and plasma samples obtained from blood. Among studies conducted to date, only one addresses TNBC status in tumorigenic patient tissue. This 2017 study included 25 TNBC and 35 healthy individuals. It was reported that *ANRIL* expression levels were higher in TNBC patients compared to the healthy group, while expression levels in tumor tissue were lower than in plasma [23].

In TNBC cells, *MALAT1* regulates cell growth and metabolism, primarily through the PI3K/Akt pathway, via the *KDM5B* (Lysine demethylase 5b) protein [24]. Dysregulation of post-transcriptional cell signaling affects mechanisms such as metastasis and response to therapy [25]. *MALAT1* is critical for TNBC because it participates in the MAPK/ERK, PI3K/Akt, and Wnt/β-catenin pathways. These pathways are directly linked to the regulation of the cell signaling network in metastasis, proliferation, and cancer development. A recently published study highlighted increased *MALAT1* expression in TNBC cell lines and suggested that it may be effective in treatment via the JAK/STAT pathway [26]. In a separate study of 88 patients who underwent mastectomy in the early stages of TNBC, high *MALAT1* expression was found in tissue samples, and it was reported that this may be associated with lymph node metastasis [27]. In our study, consistent with the literature, *MALAT1* expression levels in tissue and serum samples were higher in the patient group than in the control group. When the patient group was evaluated within itself, *MALAT1* was found to be less expressed in tissue samples than in serum samples.

First discovered for its role in hepatocellular carcinoma cells, *HULC* is known for its decreased expression levels in TNBC and other breast cancer subtypes. Studies have reported that *HULC* acts primarily through the PI3K/AKT/mTOR pathway in TNBC cells [28]. It participates in the PI3K/AKT/mTOR pathway network through MMP-2 and MMP-9 (Matrix metalloproteinase 2/Matrix metalloproteinase 9), which are calcium- and zinc-dependent proteolytic enzymes. In the literature, low *HULC* expression has been reported in studies conducted on breast cancer cell lines, plasma, and tumor tissue. In 2022, a study on an experimental animal model of cisplatin resistance reported low *HULC* expression in the metastatic TNBC group [29]. In our study, *HULC* expression, unlike other lncRNAs, was found to be associated with patient survival. Additionally, in our study, *HULC* expression was lower in the patient group than in the control group. When tissue and serum samples from the patient group were compared, expression levels were quite similar. Based on this result, *HULC* expression levels in serum samples may serve as a biomarker in TNBC.

Considering the studies conducted over the last seven years, it is interesting that the selected patient groups are not clearly defined with respect to TNBC. The patients in our study were sporadic TNBC patients. The literature lacks a clear definition of TNBC subgroups in the studies conducted. Few studies specifically address mutations in genes that influence breast cancer. The results of our study support the concept of three lncRNAs as biomarkers. When patient group tissue and patient group serum data are compared statistically, the values of the exosomal *HOTAIR*, *HULC*, and *ANRIL* genes obtained from tissue and serum appear consistent. This suggests that these three genes have biomarker value and can be studied using exosomal RNA isolated from patient serum, without invasive procedures.

Our findings, demonstrating concordant expression patterns between tissue- and serum-derived exosomal lncRNAs, are consistent with previous studies showing that circulating RNA molecules can reflect tumor tissue biology. Several studies have shown that exosomal lncRNAs in serum or plasma exhibit expression profiles comparable to those observed in tumor tissues, supporting their potential as non-invasive biomarkers in cancer diagnosis and monitoring [30,31]. These observations strengthen the reliability of serum-derived exosomal lncRNAs as surrogate markers of tumor gene expression.

The similar expression patterns of *HOTAIR*, *HULC*, and *ANRIL* observed in both tumor tissue and serum-derived exosomal samples suggest that circulating exosomal lncRNAs may partially reflect tumor-associated molecular changes. Given that exosomes carry molecular cargo derived from their cells of origin, this concordance may indicate that certain tumor-specific expression patterns are preserved in circulation.

Although these findings support the potential use of serum-derived exosomal lncRNAs as non-invasive biomarkers, they should be interpreted with caution. The consistency observed in this study provides preliminary evidence for their possible clinical relevance, particularly in TNBC, where minimally invasive approaches are of interest. However, further studies with larger cohorts are needed to validate these observations and better understand their applicability in clinical practice. The differential expression patterns observed in this study, including the upregulation of *NEAT1* and *MALAT1* and the downregulation of *HOTAIR*, *HULC*, *ANRIL*, and *AFAP1-AS1*, further support the biological heterogeneity of TNBC. These distinct expression profiles may help identify molecular subgroups and suggest the potential to develop subtype-oriented biomarker panels.

We believe that differences in some of the results obtained in our study from the literature are related to heterogeneous cohort definitions in other studies, differences in TNBC subtype distribution (luminal androgen receptor [LAR] vs. mesenchymal ratios), tissue/cell composition, and technical/standardization differences. The preferred heterogeneous cohort feature in the literature, namely the inclusion of *BRCA1/2* carriers and/or patients who have received neoadjuvant treatment, is a significant factor that increases lncRNA expression levels. In particular, the heterogeneity of tumor origins and the lack of clarity in TNBC subtype distributions in other studies directly affect the results. The prevalence of LAR tumor types in sporadic TNBC patients is low, and this low prevalence may be responsible for decreased lncRNA expression levels.

In the present study, limited associations were observed between lncRNA expression levels and clinicopathological features. Among the analyzed genes, only *NEAT1* showed a significant relationship with metastasis status, being more highly expressed in non-metastatic patients. No significant correlations were identified for the other lncRNAs or serum tumor markers.

The ROC analysis results from this study demonstrate that both tissue- and serum-derived exosomal lncRNAs have high diagnostic accuracy in distinguishing sporadic TNBC patients from healthy individuals. The near-perfect discriminatory power observed, particularly for *HOTAIR* and *ANRIL*, suggests that these molecules could be potential biomarkers for TNBC diagnosis. Similarly, the high AUC values obtained for *AFAP1-AS1*, *NEAT1*, *HULC*, and *MALAT1* also support the diagnostic performance of these lncRNAs. These consistent results in tissue and serum samples indicate that exosomal lncRNAs can carry tumor-derived molecular signals into circulation. They may therefore play an important role in non-invasive diagnostic approaches. However, due to the limited number of patients in our study and the analyses performed at a single center, the clinical potential of these biomarkers needs to be confirmed in larger, multicenter studies.

Although the ROC analysis demonstrated high diagnostic performance across all evaluated lncRNAs, these findings should be interpreted with caution due to the relatively small sample size. Therefore, the results should be considered preliminary and require validation in larger, independent cohorts. Nevertheless, these results highlight the potential of exosomal lncRNAs as promising non-invasive biomarkers in sporadic TNBC. The high diagnostic accuracy observed for certain exosomal lncRNAs, particularly *HOTAIR* and *ANRIL*, suggests their potential utility in distinguishing TNBC patients from healthy individuals. However, these findings should be interpreted with caution due to the relatively small sample size and require validation in larger cohorts before clinical application.

Additionally, the tumor microenvironment also influences lncRNA expression levels. If stromal/immune cells are abundant in the tumor microenvironment, lncRNA expression levels directly increase. Epigenetic factors, such as promoter methylation and histone modifications, can reduce gene expression in specific cell types. It is known that post-transcriptional m6A-mediated degradation mechanisms can reduce lncRNA expression, particularly in the sporadic TNBC subtype [32].

In this study, sporadic TNBC was defined as the absence of pathogenic or likely pathogenic variants identified through germline genetic testing with a hereditary breast cancer NGS panel, as well as of copy number variations (deletions/duplications) evaluated by MLPA analysis. Therefore, the classification of sporadic cases reflects the genetic testing strategy applied within this cohort. It should be noted that this definition is limited to the scope of the applied genetic testing methods and may not completely exclude all hereditary predispositions.

Therefore, it is crucial to conduct studies using subtype-stratified and composition-adjusted models, orthogonally validate selected targets with RT-qPCR, and align sub-cohorts across open datasets. Our findings reflect the biological specificity of sporadic TNBC and provide a narrower but more consistent reference for clinical subclassification.

The heterogeneous expression patterns of lncRNAs observed in this study further support the concept that TNBC represents a biologically diverse group of tumors rather than a single entity. This molecular heterogeneity may have important implications for personalized treatment strategies, as distinct lncRNA expression profiles could aid in patient stratification and the identification of clinically relevant subgroups. In this context, serum-derived exosomal lncRNAs may provide a minimally invasive approach to capture tumor heterogeneity and support more tailored therapeutic decision-making. However, further validation is required before clinical implementation.

## 4. Materials and Methods

### 4.1. Study Group

**Figure 4:**
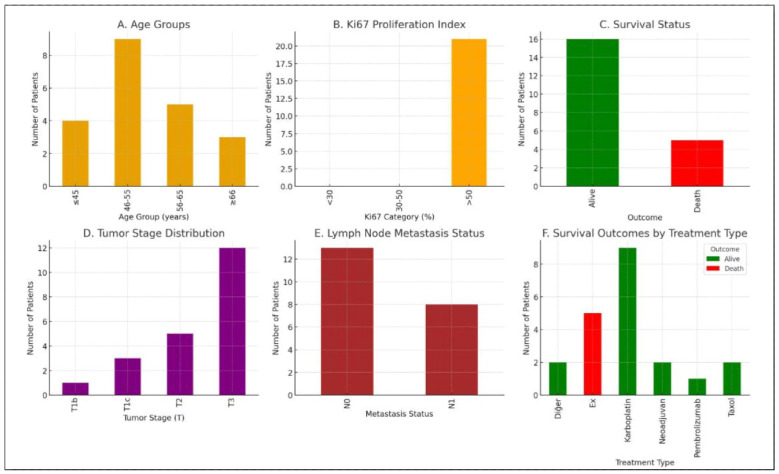
Clinical, pathological, and treatment response characteristics of the TNBC patient cohort included in the current study.

Among the patients who presented to the Departments of Medical Genetics, Department of General Surgery, Department of Radiology, and Department of Medical Oncology at Trakya University, Faculty of Medicine, with a clinical diagnosis of breast cancer between 2021 and 2025, 62 patients diagnosed with TNBC were initially screened for the hereditary breast cancer NGS panel (Qiagen Cancer Panel Kit/Hilden, Germany). Of the 62 TNBC patients, 21 (33.87%) patients with no pathogenic or likely pathogenic variants were evaluated and included in the study as having sporadic breast cancer. Tumor tissue and peripheral blood samples were collected from patients diagnosed with TNBC in two different ways. Tumor tissue samples were obtained by mastectomy by the general surgeon and by core biopsy by the radiologist (Table 4 and Figure 4).

The patient, who presented to the General Surgery Department for tissue sampling, initially had a tumor diagnosed using mammography/ultrasound techniques. After confirming the presence of the cancer, a core biopsy was performed in the Radiology Department. After histochemical analysis of the biopsy samples in the Pathology Department confirmed the presence of TNBC, a true-cut biopsy was performed using the biopsy marker for further testing.

Peripheral blood samples were collected from the patients before neoadjuvant chemotherapy treatment. Patients who underwent mammoplasty for breast reduction, etc., by a plastic, reconstructive, and esthetic surgeon were included in the healthy control group. The admission criteria for the patients and controls are listed in Table 5. When preparing patient sample data, metastasis (TNM) staging information was added to the existing information. TNM staging prioritizes primary tumor grading/breast cancer staging (T) and metastasis/lymph node involvement. The tissue and serum samples in the control group consisted of 30 healthy individuals who underwent breast reduction surgery in the Plastic Surgery Department.

### 4.2. RNA Isolation

Tissue samples from the patient and control groups were stored in RNA preservation solution at −20 °C until RNA isolation began. Blood samples from both groups were centrifuged at 3000 rpm, as was done, to separate the serum, which was stored at −80 °C. For RNA isolation from tissue samples, the Thermo-RNAqueous™ Total RNA Isolation Kit (Waltham, MA, USA) was used. To check the concentration and purity of the RNAs obtained after isolation, values in the 230–260 spectrum range were examined on the NanoDrop™ 2000/2000c device (Thermo Fisher Scientific, Waltham, MA, USA). After isolation, samples were stored at −80 °C.

Exosome isolation was performed using the Total Exosome Isolation Kit (Thermo Fisher Scientific, Waltham, MA, USA) according to the manufacturer’s protocol, which uses a polymer-based precipitation method to enrich extracellular vesicles. Following isolation, exosomal RNA and protein were extracted using the Total Exosome RNA and Protein Isolation Kit (Thermo Fisher Scientific, USA). To support the presence of exosome-enriched vesicles, commonly reported exosomal-associated proteins, including CD9, CD63, and TSG101, were evaluated within the applied workflow. Total protein concentration of isolated exosomes was quantified using the Qubit™ Protein HS Assay Kit (Thermo Fisher Scientific, USA), confirming the presence of vesicle-associated proteins. The applied methodology represents an enrichment-based approach for extracellular vesicle isolation and was performed consistently across all samples. The workflow was designed in accordance with commonly used experimental approaches in extracellular vesicle research. In addition, RNA isolated from exosomal preparations was assessed for concentration and purity using spectrophotometric methods and was successfully amplified by RT-qPCR, supporting the integrity and biological origin of the vesicular RNA. In addition, guidance was sought from researchers familiar with the MISEV2018 recommendations during the study design phase. All procedures were performed in accordance with the minimal experimental considerations for extracellular vesicle studies as outlined in the MISEV2018 guidelines [33]. After isolation, samples were stored at −80 °C.

### 4.3. Conversion of Isolated RNA Samples into Complementary DNA

Patient and control RNAs were converted to complementary DNA (cDNA) by Polymerase Chain Reaction using the Thermo Fisher-High-Capacity cDNA Reverse Transcription Kit (Waltham, MA, USA). After the reaction, the samples were stored at −20 °C.

### 4.4. Real-Time PCR Study

Gene expression of lncRNAs was studied using the Thermo Fisher Scientific TaqMan™ Non-coding RNA Assay (Waltham, MA, USA) on the Applied Biosystems StepOnePlus Real-Time PCR System. The process was performed in triplicate for each patient in a 96-well plate using specific lncRNA assays. The housekeeping gene GAPDH (Glyceraldehyde-3-phosphate dehydrogenase) was selected as the endogenous control based on its widespread use and reported stability in similar experimental settings [34]. The expression stability of GAPDH was evaluated across all analyzed samples, and Ct values showed minimal variation between groups, supporting its suitability for normalization in this study. The amplification conditions consisted of an initial denaturation step followed by 40 amplification cycles according to the manufacturer’s recommendations. Melt curve analysis was performed at the end of each run to confirm amplification specificity. Relative expression levels were calculated using the 2^−ΔΔCt^ method after normalization with the reference gene. Samples with Ct values greater than 35 were excluded from the analysis. Technical replicates with ΔCt > 1 were considered outliers and excluded from further evaluation. PCR efficiency and standard curve parameters were evaluated to ensure alignment with the Minimum Information for Publication of Quantitative Real-Time PCR Experiments (MIQE) guidelines [35].

### 4.5. Statistical Evaluation of Data

All data obtained from the study were evaluated using Mann–Whitney U and Wilcoxon W tests (Bonferroni correction/*p* < 0.001) in the *SPSS 20.0 (license: 10240642) program. The numerical data obtained from the evaluation were visualized using the *R analysis program (version 4.2.1). To determine the diagnostic performance of exome lncRNAs, ROC curve analysis was performed using IBM SPSS Statistics (SPSS 20.0; license 10240642; IBM Corp., Armonk, NY, USA). Area under the curve (AUC), 95% confidence intervals (CI), and *p*-values were calculated to assess the discriminatory ability between TNBC patients and a healthy control group. To evaluate the consistency between tissue and serum expression patterns, concordance was assessed based on the direction of expression changes (upregulation or downregulation) observed in both sample types.

## 5. Conclusions

Our study results demonstrate the biomarker potential of exosomal lncRNAs in sporadic TNBC, thus contributing significantly to the limited literature in this field. The similar expression of *HOTAIR*, *HULC*, and *ANRIL* in both tumor tissue and serum-derived exosomal lncRNAs supports the conclusion that these lncRNAs can be considered non-invasive biomarkers, particularly in sporadic TNBC patients. This has the potential to reduce the reliance on invasive procedures in the diagnosis and prognosis monitoring of sporadic TNBC.

Furthermore, the upregulation of *NEAT1* and *MALAT1*, along with the distinct expression profiles of *AFAP1-AS1*, reflects the biological heterogeneity of TNBC, contributing to a better understanding of its subtypes at the molecular level. Our study offers a unique approach by performing a comparative analysis based on both tissue and serum, unlike studies in the literature that typically focus on a single sample type (tissue or serum). In conclusion, the obtained data provide a new perspective on biomarker-based clinical management of TNBC and provide guidance for future, more comprehensive, prospective studies. We anticipate that exosomal lncRNAs may be promising biomarkers for early diagnosis, prognosis, and the development of personalized treatment strategies, particularly in subtypes such as TNBC, where targeted treatment options are limited.

## Figures and Tables

**Table 1:** Comparison of gene expression values of long non-coding RNAs between patient and control group tissue samples.

LncRNA	Patient-Median *p*-Value (Minimum/Maximum)	Control-Median *p*-Value (Minimum/Maximum)	*p*-Value (Adjust)
*HOTAIR*	0.0018 (0.0000/0.0337)	1.6179 (0.0418/11.0043)	<0.001
*HULC*	0.01038 (0.0013/0.1258)	1.224 (0.0607/15.7797)	<0.001
*ANRIL*	0.0023 (0.0002/0.0275)	1.2356 (0.1496/6.8210)	<0.001
*NEAT1*	141.0438 (2.2345/522.7582)	1.2535 (0.0170/7.7812)	<0.001
*AFAP1-AS1*	0.0117 (0.0007/0.1065)	0.8766 (0.0470/22.4711)	<0.001
*MALAT1*	18.8958 (1.8403/455.0874)	1.2017 (0.0036/20.6776)	<0.001

SPSS 20.0 (license 10240642): Mann–Whitney U and Wilcoxon W tests/Bonferroni correction.

**Table 2:** Comparison of gene expression values of long non-coding RNAs between patient and control group serum samples.

LncRNA	Patient-Median *p*-Value (Minimum/Maximum)	Control-Median *p*-Value (Minimum/Maximum)	*p*-Value (Adjust)
*HOTAIR*	0.0032 (0.0001/0.0511)	0.8861 (0.0540/6.5887)	<0.001
*HULC*	0.0080 (0.0001/0.8585)	1.1373 (0.0733/29.6508)	<0.001
*ANRIL*	0.0024 (0.0003/0.0369)	0.9797 (0.0947/6.8685)	<0.001
*NEAT1*	137.1870 (4.6267/1296.1347)	1.5773 (0.0191/9.0630)	<0.001
*AFAP1-AS1*	0.0197 (0.0012/0.1560)	0.7024 (0.0651/42.2242)	<0.001
*MALAT1*	25.9920 (1.9185/1698.4464)	1.2268 (0.0070/12.7285)	<0.001

SPSS 20.0 (license 10240642): Mann–Whitney U and Wilcoxon W tests/Bonferroni correction.

**Table 3:** Comparison of gene expression values of long non-coding RNAs between tissue and serum samples of the patient group.

LncRNA	Patient-Median *p*-Value (Minimum/Maximum)	Control-Median *p*-Value (Minimum/Maximum)	*p*-Value (Adjust)
*HOTAIR*	0.0018 (0.0000/0.0337)	0.0032 (0.0001/0.0511)	<0.001
*HULC*	0.01038 (0.0013/0.1258)	0.0080 (0.0022/0.8585)	<0.001
*ANRIL*	0.0023 (0.0002/0.0275)	0.0024 (0.0003/0.0369)	<0.001
*NEAT1*	141.0438 (2.2345/522.7582)	137.1870 (4.6267/1296.1347)	<0.001
*AFAP1-AS1*	0.0117 (0.0007/0.1065)	0.0197 (0.0012/0.1560)	<0.001
*MALAT1*	18.8958 (1.8403/455.0874)	25.9920 (1.9185/1698.4464)	<0.001

SPSS 20.0 (license 10240642): Mann–Whitney U and Wilcoxon W tests/Bonferroni correction.

**Table 4:** Detailed sample information of the patient group.

Patient Number	Age	Marker	Pathology	Tumor Location	Secondary Tumor	*MRI- Mammography/ Ultrasound	Metastasis
**1**	60	*CA15-3: 19 (0–26.2) *CEA: 4.80 (0–4.7)	*ER(-) *PR(-) *HER2-) Ki67%10	Left	None	T1b	M0/N1
**2**	55	*CA15-3: 30 (0–26.2) *CEA: 5.2 (0–4.7)	*ER(-) *PR(-) *HER2(-) Ki67%50	Left	Uterine polyp	T3	M0/N1
**3**	66	*CA15-3: 36.6 (0–26.2) *CEA: 2.82 (0–4.7) *CEA 125: 37.4 (0–35)	*ER(-) *PR(-) *HER2(-) Ki67%45	Left	None	T2	M0/N0
**4**	46	*CA15-3: 13 (0–26.2) *CEA: 3.42 (0–4.7)	*ER(-) *PR(-) *HER2(-) Ki67%30	Right	Cyst of kidney	T2	M0/N1
**5**	50	*CA15-3: 13 (0–26.2) *CEA: 3.76 (0–4.7)	*ER(-) *PR(-) *HER2(-) Ki67%47	Right	None	T3	M0/N0
**6**	45	*CA15-3: 30.6 (0–26.2) *CEA: 2.70 (0–4.7)	*ER(-) *PR(-) *HER2(-) Ki67%17	Right	None	T1c	M0/N0
**7**	61	*CA15-3: 35 (0–26.2) *CEA: 3.78 (0–4.7)	*ER(-) *PR(-) *HER2(-) Ki67%50	Right	Uterine polyp	T3	M0/N0
**8**	47	*CA15-3: 32 (0–26.2) *CEA: 3.05 (0–4.7) *CEA 125: 41 (0–35)	*ER(-) *PR(-) *HER2(-) Ki67%49	Left	None	T3	M0/N1
**9**	43	*CA15-3: 27 (0–26.2) *CEA: 2.48 (0–4.7) *CEA 125: 33.2 (0–35)	*ER(-) *PR(-) *HER2(-) Ki67%52	Right	None	T3	M0/N0
**10**	49	*CA15-3: 41.6 (0–26.2) *CEA: 2.15 (0–4.7)	*ER(-) *PR(-) *HER2(-) Ki67%30	Left	Uterine fibroids	T1c	M0/N1
**11**	45	*CA15-3: 18 (0–26.2) *CEA: 3.75 (0–4.7)	*ER(-) *PR(-) *HER2(-) Ki67%40	Left	Thyroid nodule	T3	M0/N1
**12**	52	*CA15-3: 26.1 (0–26.2) *CEA: 2.28 (0–4.7)	*ER(-) *PR(-) *HER2(-) Ki67%38	Right	None	T3	M0/N1
**13**	69	*CA15-3: 17 (0–26.2) *CEA: 2.56 (0–4.7) *CEA 125: 29 (0–35)	*ER(-) *PR(-) *HER2(-) Ki67%49	Right	None	T1c	M0/N0
**14**	45	*CA15-3: 19 (0–26.2) *CEA: 1.46 (0–4.7) *CEA 125: 37 (0–35)	*ER(-) *PR(-) *HER2(-) Ki67%29	Left	None	T3	M0/N0
**15**	63	*CA15-3: 29 (0–26.2) *CEA: 4.90 (0–4.7) *CEA 125: 33 (0–35)	*ER(-) *PR(-) *HER2(-) Ki67%57	Right	None	T3	M0/N0
**16**	55	*CA15-3: 19 (0–26.2) *CEA: 3 (0–4.7)	*ER(-) *PR(-) *HER2(-) Ki67%41	Right	None	T3	M0/N1
**17**	51	*CA15-3: 17 (0–26.2) *CEA: 4 (0–4.7) *CEA 125: 31 (0–35)	*ER(-) *PR(-) *HER2(-) Ki67%30	Left	None	T3	M0/N0
**18**	68	*CA15-3: 29 (0–26.2) *CEA: 5 (0–4.7)	*ER(-) *PR(-) *HER2(-) Ki67%47	Left	Uterine fibroids	T1c	M0/N0
**19**	56	*CA15-3: 25.7 (0–26.2) *CEA: 7 (0–4.7)	*ER(-) *PR(-) *HER2(-) Ki67%50	Left	Uterine fibroids	T2	M0/N0
**20**	65	*CA15-3: 22 (0–26.2) *CEA: 4.39 (0–4.7) *CEA 125: 33.7 (0–35)	*ER(-) *PR(-) *HER2(-) Ki67%37	Right	None	T2	M0/N0
**21**	47	*CA15-3: 26 (0–26.2) *CEA: 5.7 (0–4.7)	*ER(-) *PR(-) *HER2(-) Ki67%51	Right	None	T2	M0/N0

*CA15-3: metastatic breast cancer, *CEA: carcinoembryonic antigen, *ER: estrogen hormone receptor, *PR: progesterone hormone receptor, *HER2: epidermal growth factor receptor 2, *MRI: magnetic resonance imaging.

**Table 5:** Criteria required for patient and control group members in the current study.

Patient Group	Control Group
Female individuals between the ages of 18 and 75	Female individuals between the ages of 18 and 75
Not having a consanguineous relationship	Not having a consanguineous relationship
No pathogenic or likely pathogenic variants were detected in hereditary breast cancer-associated genes (*BRCA1*, *BRCA2*, *CHEK2*, *BRIP1*, *ATM*, *PALB2*, *P53*, *ATR*)	The individual should not have a history of cancer
Having been diagnosed with triple-negative (ER, PR, HER2-negative) breast cancer	Not having a history of breast, ovarian and endometrial cancer in first- and second-degree relatives
The conditions for not having received neoadjuvant chemotherapy were sought	

## Data Availability

The raw data supporting the conclusions of this article will be made available by the authors on request.
